# Research integrity guidelines in the academic environment: The context of Brazilian institutions with retracted publications in health and life sciences

**DOI:** 10.3389/frma.2022.991836

**Published:** 2022-10-28

**Authors:** Rafaelly Stavale, Vanja Pupovac, Graziani Izidoro Ferreira, Dirce Bellezi Guilhem

**Affiliations:** ^1^Laboratory of Bioethics, Ethics and Scientific Integrity, Postgraduate Department of Nursing, Faculty of Health and Life Science, University of Brasília, Brasília, Brazil; ^2^Department of Social Sciences and Medical Humanities, University of Rijeka, Rijeka, Croatia; ^3^Department of Social Sciences and Medical Humanities, Faculty of Medicine, University of Rijeka, Rijeka, Croatia

**Keywords:** research integrity, retracted publications, health science, research ethics, research integrity guidelines

## Abstract

Although research misconduct is responsible for most retractions in health and life sciences from authors affiliated with Brazilian institutions, there are few studies evaluating retraction notices and research misconduct in the country. Understanding the form of research misconduct may share light on the weaknesses and strengths of individual, organizational, and structural factors toward the implementation of a research integrity culture. This review on policies and practices aims to access the available information from research integrity offices and the guidelines from Brazilian funding institutions and universities who were involved in retractions in health and life science publications based on a previously published systematic review. Additionally, we summarize the available guidelines and policies for research integrity in the country. Additionally, we searched publicly available guidelines and offices for research integrity. In total, 15 institutions were analyzed: five funding agencies and 10 universities. Approximately 40% of the funding agencies promoted local research, and 60% promoted national research. Considering national funding agencies, 66% had the commission on research integrity. Approximately 30% of the universities do not have the official office for research integrity or any publicly available guidelines. Most institutions involved in retractions due to some form of research misconduct. Brazilian institutions involved in publication retractions lack instruments to prevent, supervise, and sanction research misconduct. Institutions of the country have insufficiently developed a system to promote and sustain research integrity practices. Nevertheless, there is a positive movement of researchers who are engaged in the investigation of research integrity, policy creation and training. This study emphasizes increased influence of Brazilian scientific collaboration and production globally as well as the impact of retractions in medical sciences. In contrast, it addresses the need for clear research integrity policies to foster high-quality and trustworthy research.

## Introduction

In 2018, the National Science Foundation (NSF, USA) classified countries according to their number of international publications and ranked Brazil at the 11th position, reaching the number 60,147.96 published articles (FAPESP, 2020). The country's top publications were produced in collaboration with the European Union, the USA, and the members of the BRICS cooperative group (Brazil, Russia, India, China, and South Africa; Research in Brazil, 2018). Unfortunately, a large number of medical papers have been retracted as a result of the rise in scientific production. According to Retraction Watch (RW), in the last 10 years, almost 2% (152/9,360) of all retracted health science articles are from authors affiliated with Brazilian institutions (The Retraction Watch Database, 2018). In addition, evidence has shown that the majority of medical paper retractions are related to research misconduct (Lins and Carvalho, 2014; Almeida et al., 2016; Stavale et al., 2019).

To foster the quality and reliability of research, academic leaders and institutions started to disseminate research integrity standards. A systematic review was conducted to gather all scientific articles published by Brazilian authors on research integrity (Pádua and Guilhem, 2015). This review found that the first publication on this theme dated back to 2005 and most of the articles were in the field of health and life sciences. Since then, there was an increase in the number of scientific publications about research integrity, raising awareness of the topic. However, compared to other countries, Brazil has underdeveloped system to safeguard research integrity in Brazil (Vasconcelos et al., 2015; Armond and Kakuk, 2021).

Simultaneously with the growth of publications, several articles show an increase in the number of publications, followed by concerns about the quality and reliability of these articles (Stavale et al., 2019). The first retraction reported in health and life science research from authors affiliated with Brazilian institutions was due to plagiarism (Reichembach, 2007; Stavale et al., 2019). Since then, other cases of research misconduct have been discovered, generating considerations about scientific advances in the country (Stavale et al., 2019).

In this context, this study aims to review publicly available information of research integrity policies of Brazilian institutions involved in retracted health science publications (Pádua and Guilhem, 2015).

## Research misconduct in health and life sciences

Research misconduct practices have a negative impact on public trust and favors misinformation (Armond and Kakuk, 2021). The use of unreliable findings and unethical research as a basis for future investigations is detrimental to scientific advances (Stern et al., 2014) because it affects evidence-based medicine by exposing the individual to wrong medical decisions (Steen, 2011); and may result in a waste of human and financial resources (Bordewijk et al., 2021).

Retraction notices are an important instrument for communicating that a research is flawed and unreliable (Smith, 2000). Retractions are published when honest mistakes jeopardize the reliability of a scientific work or research misconduct is found (Smith, 2000). The most common reason for retractions in health and life sciences is research misconduct (Steen, 2011; Stern et al., 2014; Stavale et al., 2019). However, the form of research misconduct that led to a retraction is controversial. A study on health and life science publications found that most of the retracted articles from researchers affiliated with Brazilian institutions were due to plagiarism (Stern et al., 2014). These findings corroborate with larger studies in South American databases (Smith, 2000). However, another study on biomedical publications concluded that the main cause of retraction was fraud (Santos-D'amorim et al., 2021).

There are few studies evaluating retraction notices and research misconduct institutions in Brazil (Pádua and Guilhem, 2015; Stavale et al., 2019; Santos-D'amorim et al., 2021). Understanding the form of research misconduct may share light to the weaknesses and strengths of individual, organizational, and structural factors toward the implementation of a research integrity culture (Haven and van Woudenberg, 2021; Santos-D'amorim et al., 2021). Research integrity policies would be more effective if they were supported by scientific evidence as they would provide a better understanding of the problem (Haven and van Woudenberg, 2021).

## Research misconduct: Definitions, inhibitors, and boosters

Scientific knowledge must be trustworthy and reliable to benefit the society (Moher et al., 2020). High research standards presume robustness, rigor, and transparency through design, execution, and reporting (ALLEA, 2017; Moher et al., 2020). These aspects are the foundation of high-quality research and research integrity principles: reliability, honesty, respect, accountability, and fairness (Smith, 2000; Resnik and Shamoo, 2011).

Practices or actions that undermine the integrity of the research or researchers would be characterized as research misconduct (ALLEA, 2017; Imperial College, 2020). Most often, data fabrication and falsification and plagiarism are accepted to jeopardize scientific knowledge; these are well-known practices of research misconduct (Smith, 2000). However, other practices, such as authorship disputes, mis-holding research results, and the lack of transparent reports, also have negative impact on the reliability of research (Smith, 2000; Imperial College, 2020). Moreover, research misconduct accounts for intentional or unintentional behaviors that damage the reliability of research. Therefore, allegation of not being aware of research integrity practices does not alleviate the impact of unethical and less rigorous practices.

Research misconduct practices have a negative impact on public trust and favors misinformation (Armond and Kakuk, 2021). In addition, they result in scientific, social, and economical waste (Steen, 2011; Stern et al., 2014; Bordewijk et al., 2021). The use of unreliable findings and unethical research as a basis for future investigations is detrimental to scientific advance (Stern et al., 2014); affects evidence-based medicine by exposing the individual to wrong medical decisions (Steen, 2011); may result in a waste of human and financial resources (Bordewijk et al., 2021). As an example, in the coronavirus disease (COVID-19) pandemic, the lack of rigor and quality of scientific studies favored the spread of fake information (Leonard et al., 2022). Misinformation during the pandemic directly affected public health policies, trust in science and, conversely, adherence to scientific recommendations to contain the disease (Leonard et al., 2022).

Research integrity guidelines play an important role in promoting research integrity principles, in addition to acting in the prevention, investigation, and application of sanctions to the cases of research misconduct (Fanelli et al., 2015; Resnik et al., 2015). Ethical guidelines and committees for research planning, conduction, and report are well-standardized in Brazil. However, specific policies for research integrity at Brazilian universities and funding agencies are scarce (Armond and Kakuk, 2021). We searched research integrity guidelines and offices of funding agencies with the highest governmental budget involved in retractions of health and life science publications (Pádua and Guilhem, 2015).

## Regulation and accountability for research misconduct of funding agencies

As for the Brazilian context, each state of the country has a State Research Foundation (SRP), which fosters scientific and technological research and dissemination. Apart from the SRP acting locally, there are national agencies to foment science, such as the Coordination of Improvement of Higher Education Personnel known as CAPES and the Brazilian National Council for Scientific and Technological Development known as CNPq.

We analyzed publicly available information of five funding agencies. Of these, 40% promoted local research locally and 60% promoted national research. Considering national funding agencies, 66% had the commission on research integrity and all published some guidelines related to research integrity practices. Still, some documents fail to contemplate principles, research misconduct, regulations, and punitive actions. For instance, CAPES published a declaration anti-plagiarism without mentioning the other aspects of research integrity. However, the institution does not have the original document at its official website. Table 1 summarizes the main Brazilian funding institutions and the available research integrity policies.

**Table 1:** Main Brazilian funding institutions and research integrity policies.

**Institution**	**Research integrity office**	**Research integrity practices**
Coordination of Improvement of Higher Education Personnel—CAPES	Unavailable	Declaration anti-plagiarism and in favor of originality (this document was not available at the agency official website at the time of this research)
National Council for Scientific and Technological Development—CNPq	Commission for research integrity	Committee for research integrity; Guidelines for research good practices
FAPESP	Technical Chamber for research ethics (CTEP): an official institutional office to act in favor or research integrity and good practices	Guidelines of academic integrity; Guidelines of research integrity and ethical responsibilities; Research integrity column at FAPESP journal; research integrity committee (São Paulo State Foundation for Research Support, 2014); Public records of ongoing investigations and updated results
FAPEMIG	Unavailable	Guidelines for responsible practices regarding rigor and integrity during scientific research
FIOCRUZ	Commission for research integrity	Guidelines for research integrity

The most active and transparent institution that promotes research integrity and responsible research is the São Paulo SRP (FAPESP). In 2011, the first guideline for research integrity in the country was published by FAPESP, giving this institution a leading position in addressing the topic. In addition, FAPESP advocates for a research integrity culture by requiring support from institutions that benefit from it to regulate the prevention, investigation, and punishment of research misconduct (Stavale et al., 2019; Moher et al., 2020).

The Brazilian National Council for Scientific and Technological Development known as CNPq has a research integrity committee that is summoned when concerns are raised with respect to research funded by the institution (Brasil, 2022). Available documents define research misconduct as plagiarism, fraud, and fabrication, and do not clearly state research integrity principles. However, it addresses the initiatives to prevent and punish research misconduct. The CNPq also had updated meetings with main researchers in the field around the country to safeguard better practices and regulations.

## The role of universities to foster research integrity

We reviewed publicly available information of authors affiliated with 10 universities with retracted publications in health sciences. To do this, we used data from our previous study (Stavale et al., 2019). An additional search was conducted to assess the total number of retracted health science publications flagged by the RW database from 2002 to 2022 (The Retraction Watch Database, 2018). While retraction notices do not only account for research misconduct, most of the universities included in this review were involved in cases of research misconduct (Stavale et al., 2019).

Two universities with the highest number of retracted publications in health and life sciences have official guidelines for good practices in research. Approximately 30% of universities involved with retracted publications do not have a research integrity office or publicly available guidelines. Table 2 summarizes the research integrity policies of Brazilian universities involved in retractions and their total number of retracted publications.

**Table 2:** Research integrity policies of Brazilian universities who are involved with retractions.

Brazilian university^*^	**Research integrity office**	**Research integrity practices**	**Number of** retractions (*N*)^**^
Universidade de São Paulo—USP	Commission of good practices in science	- Guidelines for Good Practices in Science; - Scientific Journal with a column for good practices in research	46
Universidade Federal de São Paulo—UNIFESP	Commission for institutional academic research integrity: an official commission to act against research misconduct. Created in 2017	- Guidelines for Good Practices in Science currently being elaborated. - Education program and training regarding research integrity and good practices in science; - Organization and promotion of scientific events	18
Universidade Estadual de Campinas—UNICAMP	Commission for research integrity	- Institutional policy for good practices and research integrity of the Universidade Estadual de Campinas	36
Universidade Federal do Rio de Janeiro—UFRJ	Technical Chamber for research ethics (CTEP): an official institutional office to act in favor or research integrity and good practices	- Guidelines of Academic Integrity; Guidelines of Research Integrity and Ethical Responsibilities (Camara Técnica de Ética em Pesquisa, 2021)	3
Universidade Estadual do Rio de Janeiro—UERJ	Unavailable	Unavailable	1
Universidade Federal do Rio Grande do Sul	Unavailable	- Guidelines for Responsible Practices regarding Rigor and Integrity during Scientific Research	8
Universidade Federal do Triangulo Mineiro—UFTM	Unavailable	Unavailable	3
Universidade Federal da Bahia	Unavailable	Unavailable	4
Universidade de Brasília—UnB	Unavailable	Unavailable	3
Universidade Federal do Paraná	Unavailable	Recommendations for good practices, rigor, and research integrity—formal regulation	2

* Data from articles included at previous systematic review, considering total of authors with retraction by institution regardless of authorship position on the publication. Review available at: https://doi.org/10.1371/journal.pone.0214272.

** Results from retraction watch database access at June 2022 (http://retractiondatabase.org/RetractionSearch.aspx?).

Universities play an important role as a supervisor and leader in advocating for a research integrity culture (Vasconcelos et al., 2015; Haven and van Woudenberg, 2021; Lerouge and Hol, 2022). Institutional recognition of good practices and the sanction of research misconduct favor research integrity practices. Hence, official guidelines and punitive measures, when applicable, to researchers who produce questionable research can inhibit misbehavior. Although the cases of research misconduct often cause commotion from the scientific community, universities fail to communicate the outcome of these cases with transparency. Additionally, among the institutions with the most retracted publications, there were no disciplinary actions for authors even in cases of their involvement in misconduct during multiple instances.

In Brazil, there are discrepancies in the university guidelines when it comes to the definition and penalizations of research misconduct (Vasconcelos et al., 2015). Furthermore, official research integrity agencies investigating misconduct allegations and mediating conflicts are limited. This embodies a challenge to the identification of research misconduct and the actions to tackle misbehavior.

University policies for the career progression system may also reinforce publication pressure and competitions (Haven and van Woudenberg, 2021; Santos-D'amorim et al., 2021). These behaviors are prejudicial to research integrity principles (Stavale et al., 2019).

Although not all the universities involved in retracted publications had guidelines and research integrity offices, the number of scientific integrity committees and commissions in the country has grown significantly. For instance, the Federal University of Goiás, the Federal University of São Paulo, the Federal University of ABC, and the Instituto Israelita de Ensino e Pesquisa Albert Einstein have specific scientific research offices.

## Current challenges and future steps

In recent years, Brazilian universities, funding agencies, and researchers have been advocating for a research integrity culture. In 2010, the first Brazilian Meeting on Research Integrity and Publication Ethics (BRISPE) took place. The event was the result of a postdoctoral research project on research integrity at UFRJ (Santos-D'amorim et al., 2021). It was an important step toward research integrity awareness (Santos-D'amorim et al., 2021).

Considering funding agencies, in 2011, FAPESP was a pioneer in publishing official policies for research integrity practices. The funding agency initiative encouraged other institutions to follow. As for research institutions, most southern universities had some guidelines or the commission on research integrity (Research in Brazil, 2018). These universities account for more research aids, publications, and national and international collaborations throughout the country.

In recent years, Brazilian researchers and institutions played a leadership role into research integrity safeguards and investigations (Santos-D'amorim et al., 2021). Still, there is a long road ahead. Standardized guidelines and policies for research integrity that address research misconduct are not yet available in all institutions (Vasconcelos et al., 2012).

Most of the publications from Brazilian institutions were produced in collaboration with different countries (Research in Brazil, 2018). Hence, accountability concerning scientific reliability and quality of Brazilian institutions should be extended to those partnerships. The recognition of the significant contribution of Brazilian research institutions to the scientific community is fundamental to encourage cooperation toward the creation of research integrity policies and guidelines that are appropriate for the country context. Yet, these guidelines and policies for research integrity should comply with international standards (Desmond and Dierickx, 2021). Nevertheless, it should consider social, cultural, and institutional diversity to warrant reliability, fairness, and equity (Vasconcelos et al., 2012).

**Figure 1:**
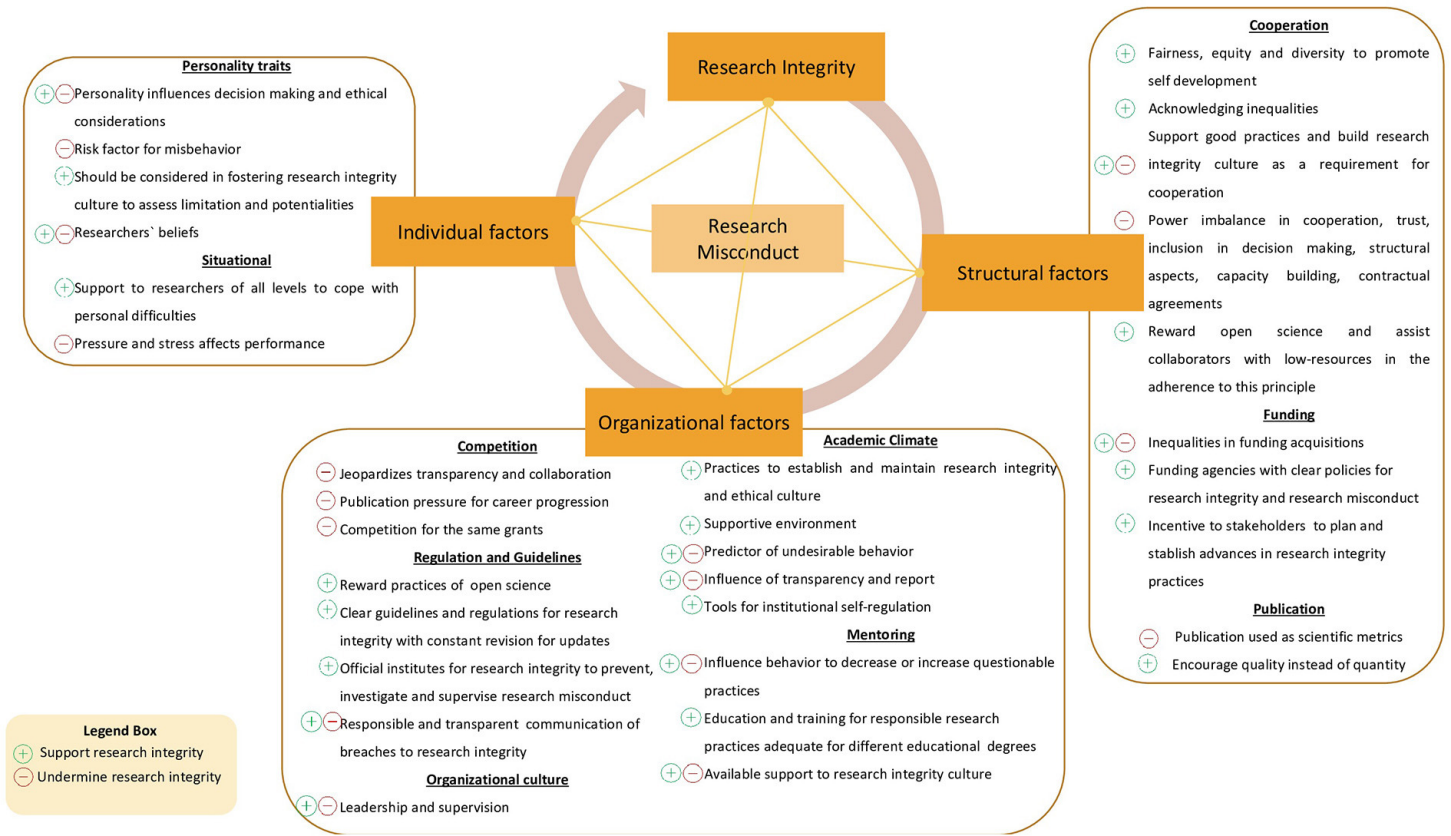
Research integrity factorize the network that influence misconduct.

Several aspects may contribute or inhibit research misconduct practices (Haven and van Woudenberg, 2021). It is known that it involves a complex and dynamic network of aspects that engage individual to structural action (Davis et al., 2007; Figure 1). Furthermore, Brazil has limitations at the organizational level (academic climate, mentoring, guidelines, and competition) and the systemic level (cooperation, funding, and publication) network that supports research integrity. More studies are needed to understand the individual level characteristics of researchers affiliated with Brazilian institutions.

At the organizational level, academic climate is undervalued. Although there are standardized tools to evaluate academic climate, there are no available publications with the validated instrument or assessing academic climate in the country (Mumford et al., 2007; Wells et al., 2014). Hence, the self-regulation of institutions is flawed.

Evidence showed that mentoring in research integrity may or may not influence positive behavior (Fanelli et al., 2015). In Brazil, universities and funding agencies foment education and training to promote research integrity practices (Vasconcelos et al., 2015; Armond and Kakuk, 2021). However, more institutions across the country must be involved in the theme. In addition, the shortage of standardized recommendations in the country can be a challenge for researchers and educators.

At a systemic level, researchers often struggle to adhere to research integrity principles due to limited financial resources, lack of infrastructure, competitive environment, or inadequate training. Brazil collaborates with institutions of higher and lower research capacity compared to its own institutions (Research in Brazil, 2018). Nevertheless, the cooperation among scientific institutions with unequal resources should consider balancing potentialities and fragilities to build trust and increase a research integrity culture (Schroeder et al., 2019; Horn et al., 2022).

## Limitations and strengths

This review of policies and practices considered the information available at the institutional website; therefore, we cannot rule out the possibility of existing outdated documents available online or the existence of non-included documents. However, up-to-date online information on the websites of official institutions promotes transparency and information accessibility. This study highlights the importance of research integrity guidelines, committees, and ongoing investigations to be made available at public official websites.

## Conclusion

Brazilian institutions involved in retracted publications lack instruments to prevent, supervise, and sanction research misconduct. Although Brazil has an increasing influence in health research globally, Brazilian institutions have pieces of what should be a system to promote and sustain research integrity practices. Still, there is a positive movement of researchers who are engaged in the investigation of research integrity, policy creation and training.

We recommend future policies and guidelines to follow international standards with respect to research reliability, fairness, diversity, and equity. The network of individual, organizational, and structural factors that can affect research integrity is useful to understand misbehavior and to guide future action. In addition, specific grants for research integrity investigations would accelerate awareness and increase resources to foster responsible research in the country.

At last, a collaboration with international institutions should consider equal accountability for research misconduct. The impact of unreliable science is borderless and demands global action to support a research integrity culture.

## Author contributions

RS conceived the presented idea and developed and experimented this study. GF and DG verified the analytical methods. VP encouraged to investigate a new specific aspect and supervised the results of this work. All authors respond to the results and to the final manuscript.

## Funding

This work was funded by the Coordination for the Improvement of Higher Education Personnel - CAPES Brazil (grant number: 1651856).

## Conflict of interest

The authors declare that the research was conducted in the absence of any commercial or financial relationships that could be construed as a potential conflict of interest.

## Publisher's note

All claims expressed in this article are solely those of the authors and do not necessarily represent those of their affiliated organizations, or those of the publisher, the editors and the reviewers. Any product that may be evaluated in this article, or claim that may be made by its manufacturer, is not guaranteed or endorsed by the publisher.
