# Supplementary material for: Research integrity guidelines in the academic environment: The context of Brazilian institutions with retracted publications in health and life sciences
Source: Front Res Metr Anal. 2022 Oct 28;7:991836. doi: 10.3389/frma.2022.991836 (PMC9650250; doi:10.3389/frma.2022.991836)
Supplement: Supplementary file 1 [file Table_2.doc]

SUPPLEMENTARY DATA

| **University** | **State** | **Identified documents** | **Search at Retraction watch** | **Total of retracted publications***  **(N)** | **Authors with retracted publications** (N)** |
| --- | --- | --- | --- | --- | --- |
| Universidade de São Paulo - USP | SP | -Guia de Boas Práticas Científicas – USP – 2019  -Portaria PRP n' 595 de 29 de agosto de 2017 | Universidade de São Paulo OR USP OR University of São Paulo OR São Paulo University | 46 | 74 |
| Universidade Federal de São Paulo - UNIFESP | SP | - [Código de Boas Práticas Científicas](https://www.unifesp.br/campus/gua/images/Biblioteca/FAPESP-Codigo_de_Boas_Praticas_Cientificas.pdf)  - Escritório de integridade acadêmica | Universidade Federal de São Paulo OR UNIFESP OR Federal University of São Paulo | 18 | 0 |
| Universidade Estadual de Campinas - UNICAMP | SP | - Política Institucional de Boas Práticas e Integridade em Pesquisa da Unicamp  -Política Institucional de Acesso Aberto à Produção Intelectual e Científica da Unicamp | Universidade Estadual de Campinas OR UNICAMP OR University of Campinas | 36 | 123 |
| Universidade Federal do Rio de Janeiro - UFRJ | RJ | -Diretrizes sobre integridade acadêmica, UFRJ  -Fluxograma preliminar – Integridade em Pesquisa | Universidade Federal do Rio de Janeiro OR UFRJ OR Federal University of Rio de Janeiro | 3 | 2 |
| Universidade Federal do Rio Grande do Sul - UFRGS | RS | - Guia para integridade em pesquisa cientifica | Universidade Federal do Rio Grande do Sul OR UFRGS OR Federal University of Rio Grande do Sul | 8 | 7 |
| Universidade Federal do Triangulo Mineiro- UFTM | MG | Unavailable | Universidade Federal do Triangulo Mineiro OR UFTM OR Federal University of Triangulo Mineiro | 3 | 3 |
| Universidade Estadual do Rio de Janeiro - UERJ | RJ | Unavailable | State University of Rio de Janeiro OR UERJ OR Universidade do Estado do Rio de Janeiro OR State University Hospital of Rio de Janeiro | 1 | 4 |
| Universidade Federal da Bahia - UFBA | BA | Unavailable | Universidade Federal da Bahia OR UFBA OR Federal University of Bahia | 4 | 8 |
| Universidade Federal do Paraná -UFPR | PR | -Instrução normativa nº 02 –CPDCT/PRPPG/UFPR para boas práticas, integridade cientifica e rigor | Universidade Federal do Paraná OR UFPR OR Federall University of Paraná | 2 | 2 |
| Universidade de Brasília - UnB | DF | Unavailable | Universidade de Brasília OR UnB OR University of Brasília OR Brasília University | 3 | 9 |
| **Funding Agency** | **Activity** | **Identified documents** | **Observations** | | |
| Coordination of Improvement of Higher Education Personnel - CAPES | National | Unavailable | Orientações CAPES de combate ao Plágio, mencionado por outros documentos | | |
| National Council for Scientific and Technological Development - CNPq | National | - Relatório da Comissão de Integridade de Pesquisa do CNPq  - Relatório da 7ª Reunião da Comissão  -Resolução Normativa Nº: 006/2012  -Portaria Nº: 091/2012 |  | | |
| FAPESP | Local -SP state | -Código de Boas Práticas Científicas FAPESP em inglês, português e espanhol -Portaria PR nº 09/2013 - Dispõe sobre a inclusão de cláusula de compromisso de boas práticas em Acordos e Convênios celebrados pela FAPESP com pessoas jurídicas privadas-Portaria PR Nº 05/2013 - Dispõe sobre a divulgação das práticas de más condutas científicas apuradas pela FAPESP |  | | |
| FAPEMIG | Local – MG state | -Plano de integridade da FAPEMIG |  | | |
| FIOCRUZ | National | Guia de Integridade em Pesquisa da Fiocruz  - Proposta de Fluxo para a Comissão de Integridade em Pesquisa |  | | |
| *Results from retraction watch database access at June 2022 (http://retractiondatabase.org/RetractionSearch)  **Data from articles included at previous systematic review, considering total of authors with retraction by institution regardless of authorship position on the publication. Review available at: https://doi.org/10.1371/journal.pone.0214272 | | | | | |
